# Supplementary figures and images for: VALERIE: Visual-based inspection of alternative splicing events at single-cell resolution
Source: PLoS Comput Biol. 2020 Sep 8;16(9):e1008195. doi: 10.1371/journal.pcbi.1008195 (PMC7500686; doi:10.1371/journal.pcbi.1008195)

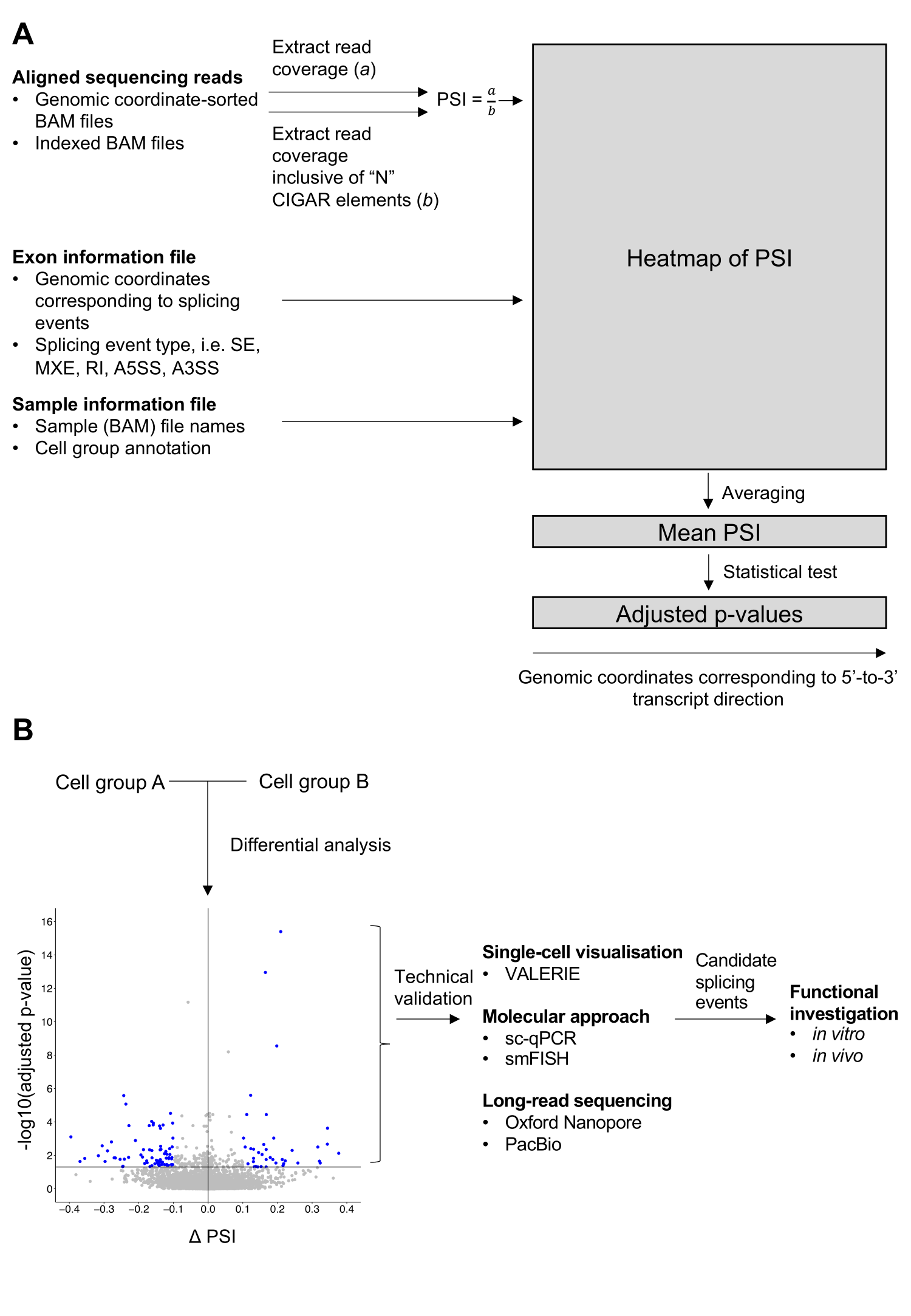

Supplement: S1 Fig — (A) The workflow of data processing steps. VALERIE computes percent spliced-in (PSI) values from read coverage information and integrates alternative splicing coordinates and cell group annotations from exon and sample information files to generate heatmap of PSI, and line graphs of mean PSI and adjusted p-values at each nucleotide position. (B) The role of VALERIE in the overall process of identifying candidate alternative splicing events for downstream functional studies. VALERIE serves as a visual inspection and validation of alternative splicing events identified from genome-wide analysis such as differential analysis. In conjunction with, or as an alternative to, other technical validation approaches as such sc-qPCR, VALERIE can enable selection of candidate alternative splicing events for downstream functional validation. A3SS: Alternative 3’ splice site. A5SS: Alternative 5’ splice site. MXE: Mutually exclusive exons. RI: Retained-intron. sc-qPCR: Single-cell quantitative polymerase chain reaction. SE: Skipped-exon. smFISH: Single-molecule fluorescence in situ hybridisation. (TIFF) [file pcbi.1008195.s001.tiff]

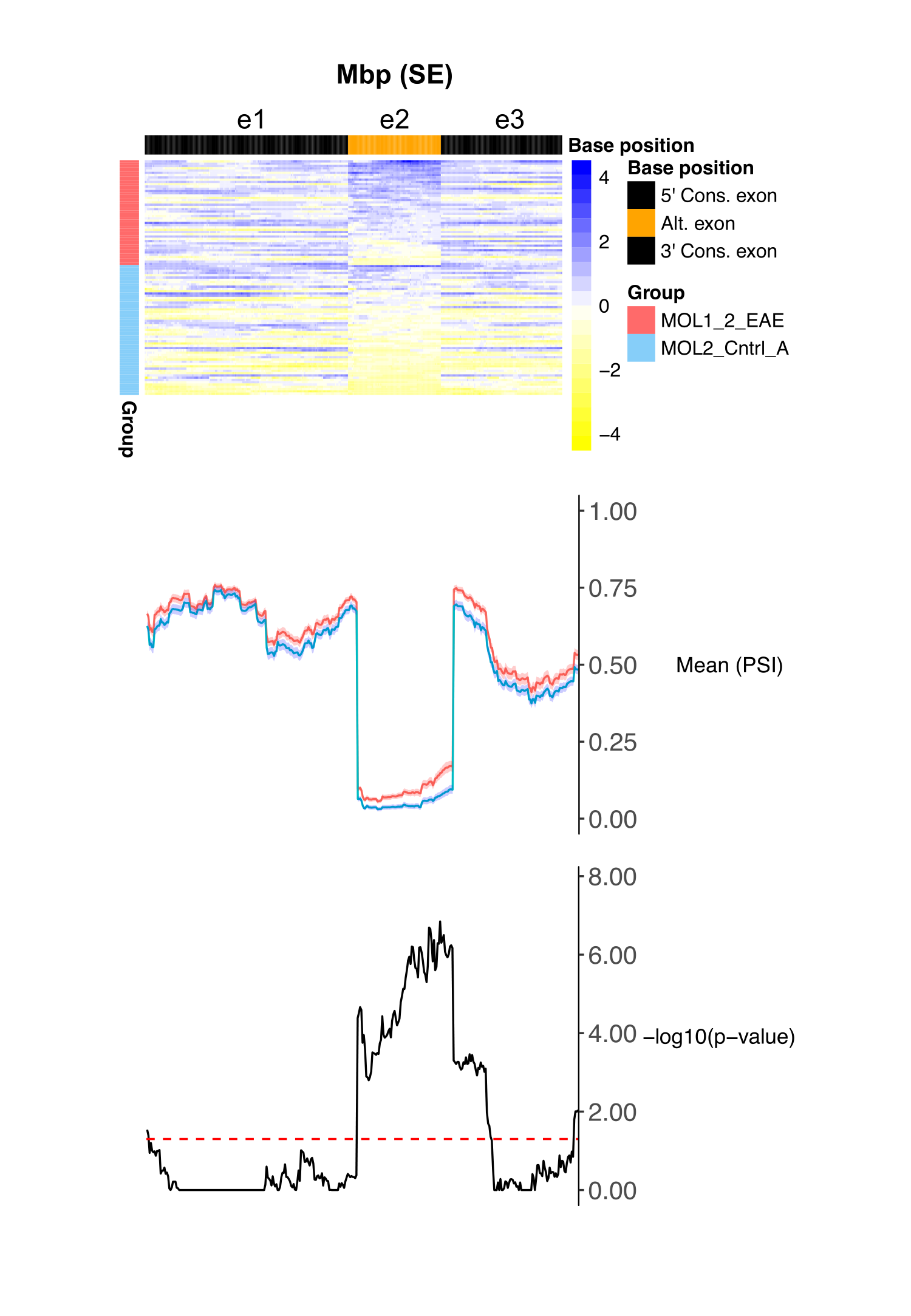

Supplement: S2 Fig — Top: Sparse profile of PSI values due to long exon lengths relative to sequencing reads. Here, exon 1, 2, and 3 are 171, 78, and 102 base-pair (bp) in length whereas libraries were sequenced in 50bp single-end mode. Middle: Mean PSI values across the genomic coordinates corresponding to the flanking constitutive exons and skipped-exon. Overall, single cells from EAE mice showed increased exon 2 usage compared to single cells from control mice. Bottom: Differences in mean PSI values across EAE and control cell groups were statistically significant at the genomic coordinates corresponding to alternative splicing event (skipped-exon) but were, as expected, not statistically significant at the genomic coordinates corresponding to the flanking constitutive exons. P-values were computed using Wilcoxon rank-sum test and adjusted for multiple testing using Bonferroni correction. The red dashed line indicates −log10 of the p-value of 0.05. Colour bar indicates scaled PSI values (z-scores) across rows (single cells). Grey regions in the heatmap indicate genomic positions with less than 10x coverage. Alt. exon: Alternatively spliced exon. Cons. exon: Constitutive exon. SE: Skipped-exon. (TIFF) [file pcbi.1008195.s002.tiff]

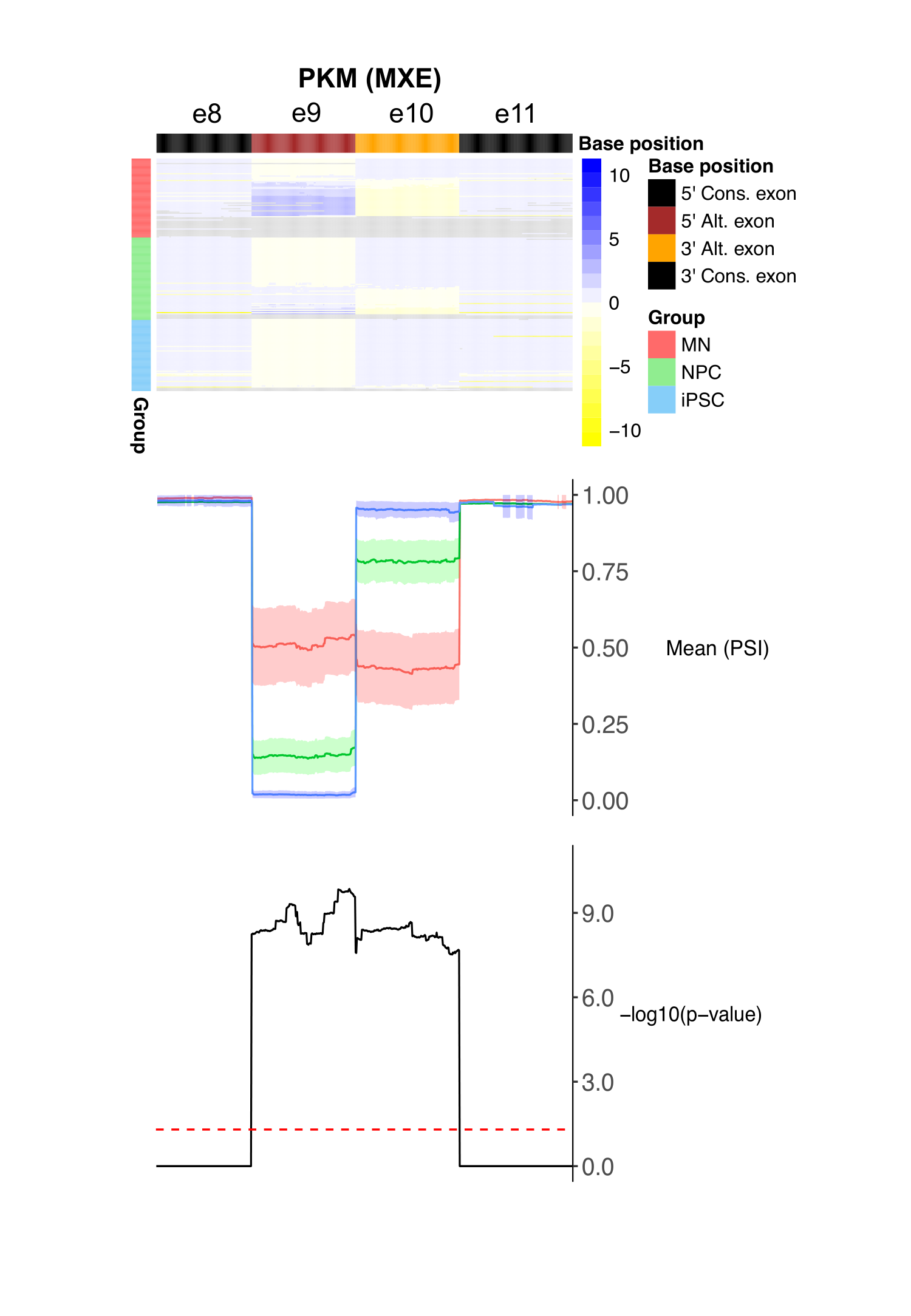

Supplement: S3 Fig — Sequencing reads were aligned using HISAT2 [29] in lieu of STAR [28] as in Fig 1. Alignment with HISAT2 similarly showed significant differential PKM mutually exclusive exon usage across the three cell populations. P-values were computed using Kruskal-Wallis test and adjusted for multiple testing using Bonferroni correction. The red dashed line indicates −log10 of the p-value of 0.05. Colour bar indicates scaled PSI values (z-scores) across rows (single cells). Grey regions in the heatmap indicate genomic positions with less than 10x coverage. Alt. exon: Alternatively spliced exon. Cons. exon: Constitutive exon. MXE: Mutually exclusive exons. (TIFF) [file pcbi.1008195.s003.tiff]

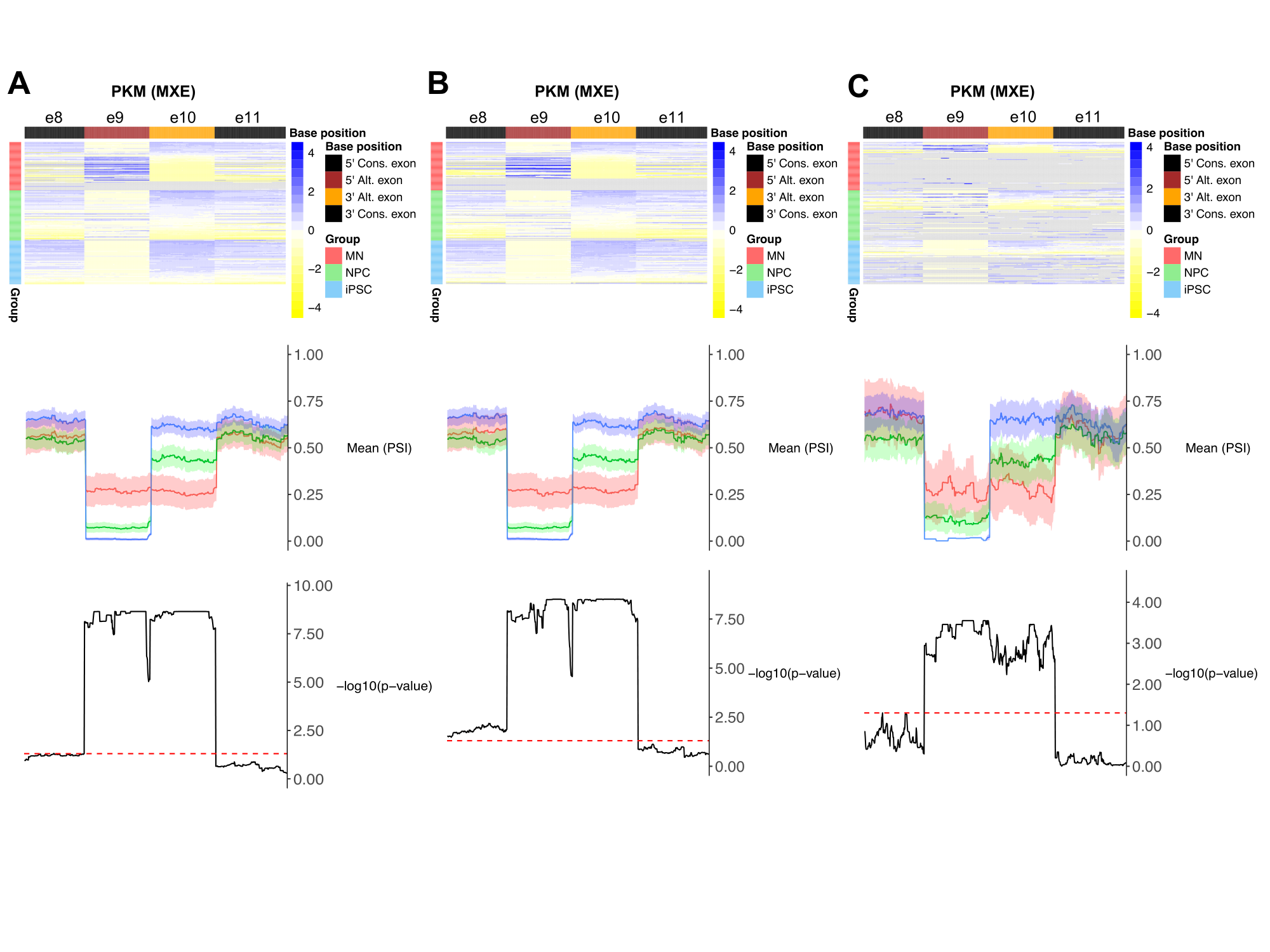

Supplement: S4 Fig — Aligned sequencing reads were subsampled to yield read depth of (A) 50%, (B) 25%, and (C) 1% of the original read depth to simulate PSI profile at different read depth. P-values were computed using Kruskal-Wallis test and adjusted for multiple testing using false discovery rate (FDR). The red dashed line indicates −log10 of the p-value of 0.05. Colour bar indicates scaled PSI values (z-scores) across rows (single cells). Grey regions in the heatmap indicate genomic positions with less than 10x coverage. Alt. exon: Alternatively spliced exon. Cons. exon: Constitutive exon. MXE: Mutually exclusive exons. (TIFF) [file pcbi.1008195.s004.tiff]

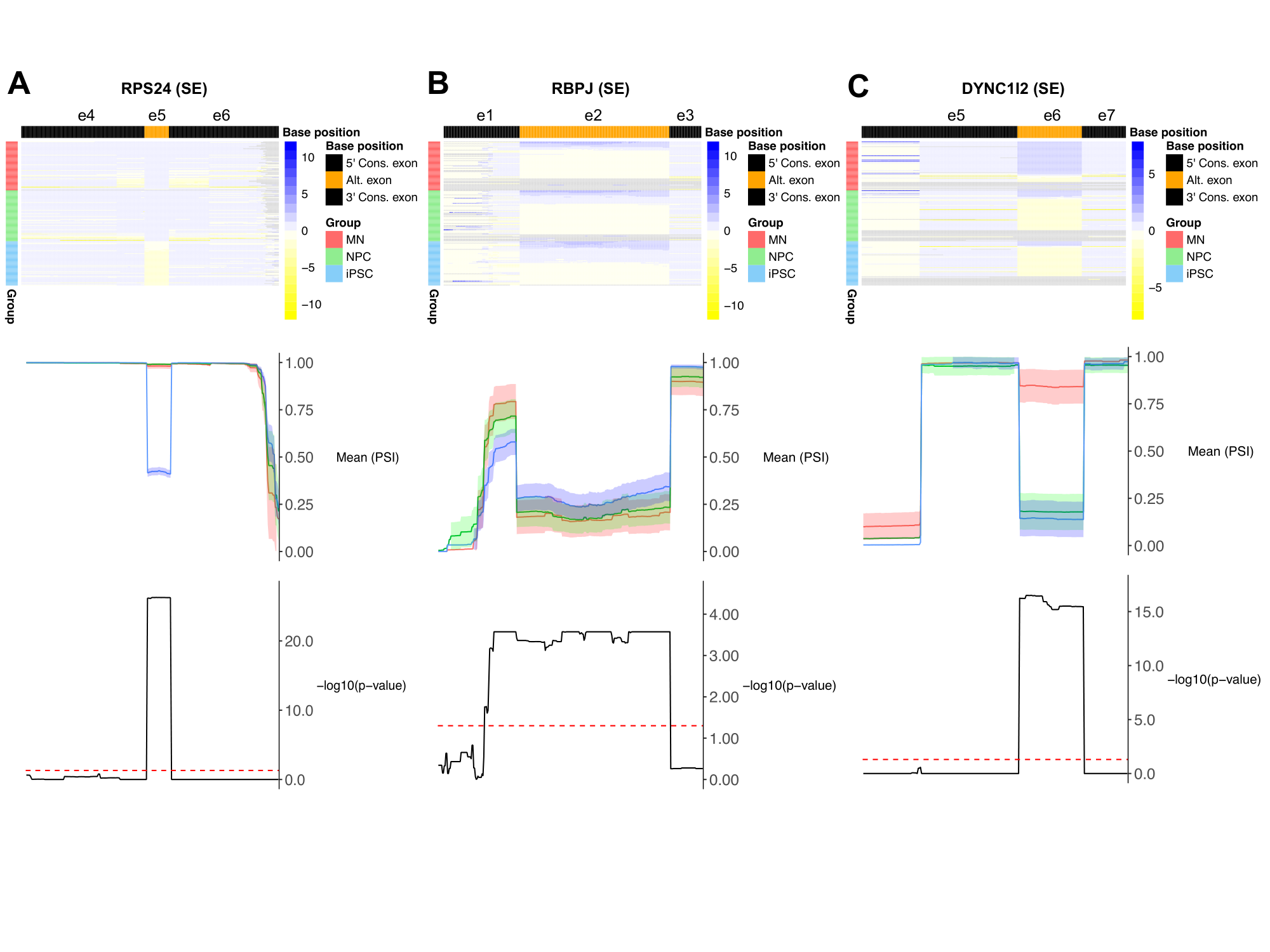

Supplement: S5 Fig — (A) RPS24 alternatively spliced exon positioned at the 3’-end (2nd last exon) of the ENST00000435275.5_4 transcript consisting 6 exons in length. (B) RBPJ alternatively spliced exon positioned at the 5’-end (2nd exon) of the ENST00000355476.7_2 transcript consisting 12 exons in length. (C) DYNC1I2 alternatively spliced exon positioned in the middle (6th exon) of the ENST00000355476.7_2 transcript consisting 18 exons in length. Differences in PSI values on the DYNC1I2 5’ constitutive exon (exon 5) revealed an alternative 3’ splice site (A3SS) located on the exon. This A3SS is annotated in GENCODE. Transcript IDs correspond to GENCODE v34lift37. P-values were computed using Kruskal-Wallis test and adjusted for multiple testing using Bonferroni correction. The red dashed line indicates −log10 of the p-value of 0.05. Colour bar indicates scaled PSI values (z-scores) across rows (single cells). Grey regions in the heatmap indicate genomic positions with less than 10x coverage. Alt. exon: Alternatively spliced exon. Cons. exon: Constitutive exon. SE: Skipped-exon. (TIFF) [file pcbi.1008195.s005.tiff]

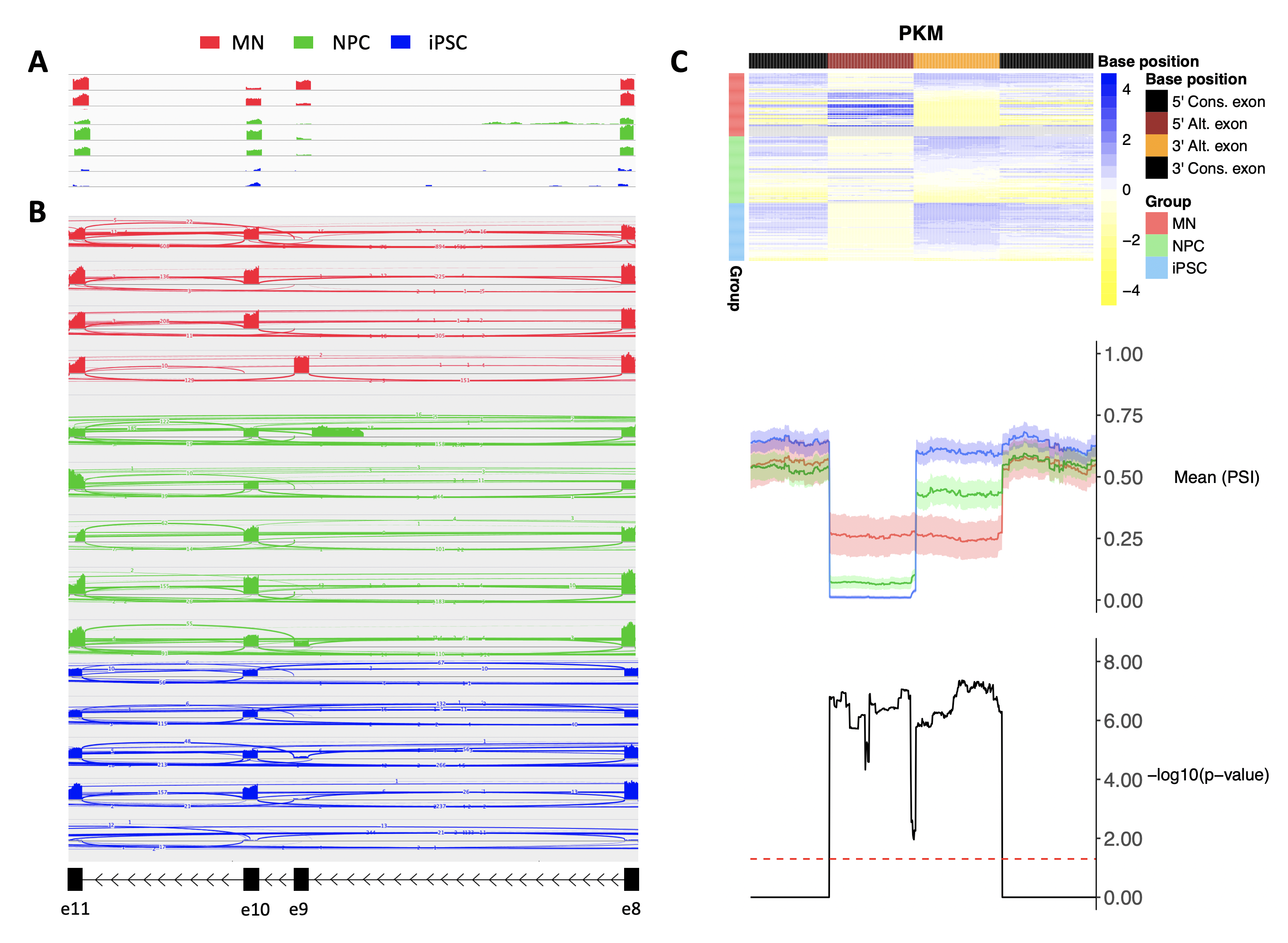

Supplement: S1 File — (GZ) [file pcbi.1008195.s006.gz › S1/VALERIE-master/inst/extdata/Plots/2_MXE_Plots_PKM.png]

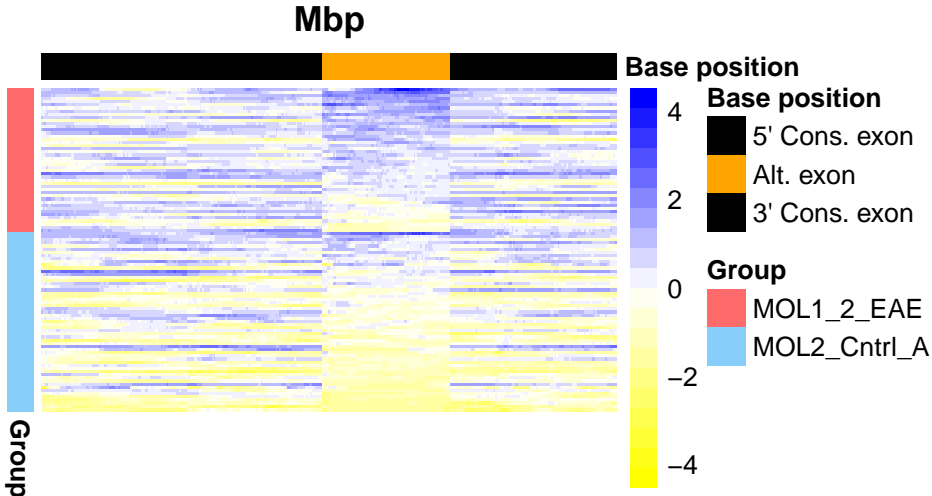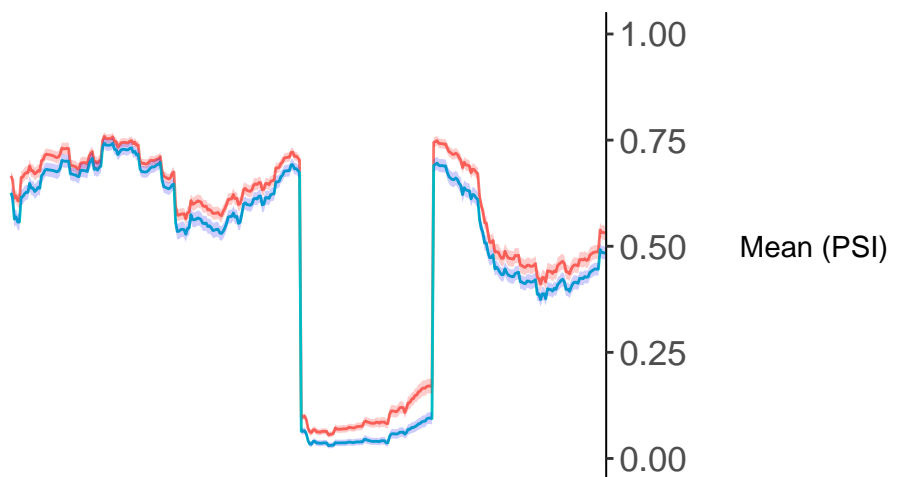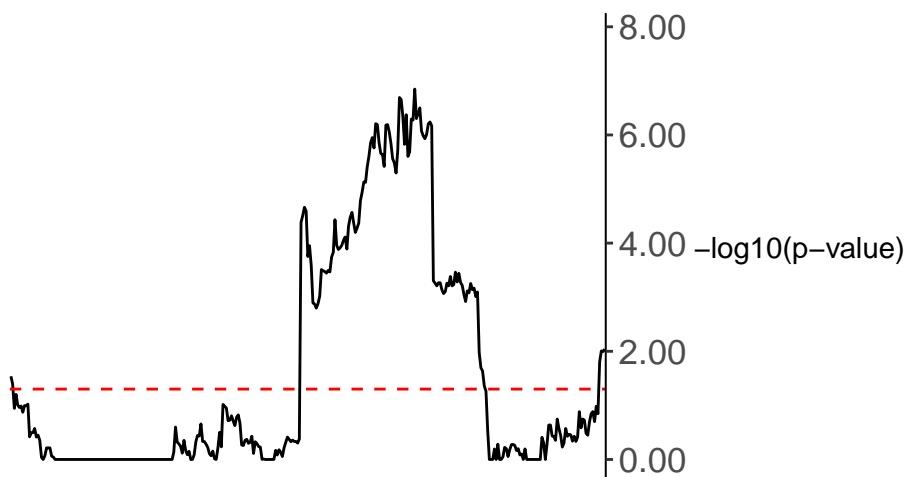

Supplement: S1 File — (GZ) [file pcbi.1008195.s006.gz › S1/VALERIE-master/inst/extdata/Plots/1_SE_Plots_Mbp.pdf]
