# Supplementary material for: VALERIE: Visual-based inspection of alternative splicing events at single-cell resolution
Source: PLoS Comput Biol. 2020 Sep 8;16(9):e1008195. doi: 10.1371/journal.pcbi.1008195 (PMC7500686; doi:10.1371/journal.pcbi.1008195)
Supplement: S1 File — (GZ) [file pcbi.1008195.s006.gz › S1/Test Data/VALERIE.pdf]

VALERIE

Sean Wen

2020-04-03

Introduction

Alternative splicing enables multiple transcripts or isoforms to arise from a gene, consequently increasing functional diversity of a gene. A notably example is Bcl-x gene. Bcl-x(L) splice variant has anti-apoptotic activity whereas Bcl-x(S) splice variant has pro-apoptotic activity [1]. To date, single-cell alternative splicing have been primarily studied in cells of the nervous and immune system [2, 3]. Current genome browsers are optimized to visualize gene expression profiles generated from small-scale bulk RNA-sequencing experiments [4]. This strategy obscure or do not capture cell-to-cell heterogeneity in alternative splicing profiles in single cell populations. Therefore, there remains a need for visualization platforms to visualize alternative splicing events at single-cell resolution. To this end, we develop VALERIE (*V*isulazing *AL*ternative splicing *E*vents from *RI*bonucleic acid *E*xperiments) - a visualization platform to address the challanges in visualizing alternative splicing events at single-cell resolution. Key features of VALERIE include:
(1) Displays PSI instead of conventional coverage/expression.
(2) Ability to scale to large datasets consisting of hundreds or even thousands of samples typical of single cell experiments.
(3) Summarizes PSI profile for user-defined groups of single cells.
(4) Assess statistical significance of PSI profiles between groups of single cells.
(5) Omits non-informative intronic regions. (6) Standardizes genomic coordinates from 5' to 3' transcription direction.

VALERIE is designed for visualizing alternative splicing events from short-read scRNA-seq data. Therefore, visualization is restricted to exon-level alternative splicing events, as opposed to full-length isoform expression. Exon-level alternative splicing events primarily encompass skipped-exon (SE), mutually exclusive exons (MXE), retained intron (RI), alternative 5' splice site (A5SS), and alternative 3' splice site (A3SS).

Design

At each genomic coordinate spanning the alternative spliced exon and its flanking constitutive exon(s), *GenomicAlignments* was used to tabulate number of reads with non-N CIGAR operation and total number of reads. Total number of reads is the sum of reads with non-N CIGAR operation and reads with N-CIGAR operation. Reads with non-N CIGAR operation are complete (non-split reads) whereas reads with N CIGAR operation are split reads and indicate splicing events. PSI values are computed by taking the number of reads with non-N CIGAR operation and dividing it by the total number of reads. Next, the PSI values for every single cell are plotted in the form of a heatmap using *pheatmap*. The PSI values at each genomic coordinate for each group of single cells are summarized using the mean and the corresponding p-value is determined. P-values can be assess using student t-test or wilcoxon rank-sum test for 2-group comparison or ANOVA or Kruskal-Wallis test for 3-group comparison. The means and p-values at each genomic coordinate are then presented in a line graph using *ggplot2*. Gene structures are present to indicate the location of the alternative exon relative to its flanking constitutive exon(s).

Installation

```r
library(devtools)
install_github("wenweixiong/VALERIE")
```

Load package

```
library(VALERIE)
```

Example data

The example data used here were from a previous publication [5]. In this study, scRNA-seq was performed on single cells obtained from the spinal cords of mice induced with experimental autoimmune encephalomyelitis (EAE) and untreatment mice serving as controls. The library preparation accomplished using Smartseq-2 and then subjected to 50bp single-end sequencing [6]. BRIE, a computational tool for infering PSI values based on sequencing reads and sequence features, was used to identify significant alternative splicing events between the two groups of single cells. Subsequently, Mbp was found to be alternatively spliced between the two groups of single cells. Specifically, Mbp exon 2 was found to have higher PSI values in EAE compared to control mice. This spling event was independently validated in a subsequent experiment using quantitative polymerase chain reaction (qPCR). Here, we will demonstrate the visual-based validation of this splicing event using VALERIE.

Three file types are required.
(1) **ExonInfo** file: Tab-delimited file describing the alternative splicing events. First column contains the alternative splicing nomenclature generated by BRIE [7] or MISO [8]. Second column indicates the type of alternative splicing event, namely SE, MXE, RI, A5SS, and A3SS. Third column contains the gene name or any personal notation. This notation will be used as the main title of plot and output plot names.
(2) **SampleInfo** file: Tab-delimited file describing the naming and grouping of the single cells. First column should contain the names of the binary alignment map (BAM) files. Second column indicates the grouping for each single cell, i.e. Group1 and Group2. Third column indicates the group names, e.g. treatment and control. The group names here for EAE and control mice single cells are MOL1_2_EAE and MOL2_Cntrl_A, respectively.
(3) **BAM** files: Binary alignment map (BAM) files sorted by genomic coordinates.

```
# Exon file (General)
exon.info <- read.table(system.file("extdata/Exon_Info", "Exon_Info_Further_Examples.txt",
    package = "VALERIE"), sep = "\t", header = FALSE, stringsAsFactors = FALSE)
print(exon.info)
#>                                                                                          V1
#> 1                                 chr5:154858639:154858768:+@chr5:154859695:154859873:+@chr5:154863207:154863395
#> 2                                  chr13:30462538:30462712:-@chr13:30461639:30461688:-@chr13:30456704:30461533
#> 3      chr15:78542177:78542219:+@chr15:78542483:78542645:+@chr15:78544190:78544267:+@chr15:78544869:78544957:+.B
#> 4  chr5:135360497:135360607:-@chr5:135352946:135353045:-@chr5:135350823:135350913:-@chr5:135345968:135346057:-.B
#> 5                                                             chr12:6536684:6536817:+@chr12:6536920:6537010:+.A
#> 6                                                           chr1:25245301:25245150:-@chr1:25244224:25243550:-.A
#> 7                                             chr11:68032286:68032336|68032523:+@chr11:68032923:68033012:+.A
#> 8                                             chr7:106092669:106092548|106092564:-@chr7:106090503:106091939:-.A
#> 9                                                  chr6:29944122:29944397:+@chr6:29944500|29945059:29945091:+.B
#> 10                                                 chr6:31269966:31270085:-@chr6:31269525|31269543:31269493:-.A
#>      V2     V3
#> 1    SE  CNOT8
#> 2    SE  HMGB1
#> 3   MXE  PSMA4
#> 4   MXE  H2AFY
#> 5    RI  GAPDH
#> 6    RI  RSRP1
#> 7  A5SS NDUFS8
#> 8  A5SS  SYPL1
#> 9  A3SS  HLA-A
#> 10 A3SS  HLA-C

# Exon file (Use for this example)
exon.info <- read.table(system.file("extdata/Exon_Info", "Exon_Info.txt",
    package = "VALERIE"), sep = "\t", header = FALSE, stringsAsFactors = FALSE)
print(exon.info)
#>                                                                                V1
#> 1 chr18:82554580:82554750:+@chr18:82561778:82561855:+@chr18:82572825:82572926
#>   V2  V3
#> 1 SE Mbp

# Sample information file
sample.info <- read.table(system.file("extdata/Sample_Info",
    "Sample_Info.txt", package = "VALERIE"), sep = "\t", header = FALSE,
    stringsAsFactors = FALSE)
head(sample.info)
#>                                                           V1     V2
#> 1 SRR7103969_STAR_aligned_2ndPass_Aligned.sortedByCoord.out.bam Group1
#> 2 SRR7103974_STAR_aligned_2ndPass_Aligned.sortedByCoord.out.bam Group1
#> 3 SRR7103980_STAR_aligned_2ndPass_Aligned.sortedByCoord.out.bam Group1
#> 4 SRR7103993_STAR_aligned_2ndPass_Aligned.sortedByCoord.out.bam Group1
```

```r
#> 5 SRR7103995_STAR_aligned_2ndPass_Aligned.sortedByCoord.out.bam Group1
#> 6 SRR7103997_STAR_aligned_2ndPass_Aligned.sortedByCoord.out.bam Group1
#>           V3
#> 1 MOL1_2_EAE
#> 2 MOL1_2_EAE
#> 3 MOL1_2_EAE
#> 4 MOL1_2_EAE
#> 5 MOL1_2_EAE
#> 6 MOL1_2_EAE
tail(sample.info)
#>                                                                  V1     V2
#> 98  SRR7104861_STAR_aligned_2ndPass_Aligned.sortedByCoord.out.bam Group2
#> 99  SRR7104972_STAR_aligned_2ndPass_Aligned.sortedByCoord.out.bam Group2
#> 100 SRR7105011_STAR_aligned_2ndPass_Aligned.sortedByCoord.out.bam Group2
#> 101 SRR7105040_STAR_aligned_2ndPass_Aligned.sortedByCoord.out.bam Group2
#> 102 SRR7105057_STAR_aligned_2ndPass_Aligned.sortedByCoord.out.bam Group2
#> 103 SRR7105061_STAR_aligned_2ndPass_Aligned.sortedByCoord.out.bam Group2
#>              V3
#> 98  MOL2_Cntrl_A
#> 99  MOL2_Cntrl_A
#> 100 MOL2_Cntrl_A
#> 101 MOL2_Cntrl_A
#> 102 MOL2_Cntrl_A
#> 103 MOL2_Cntrl_A

# BAM files
BAM <- system.file("extdata/BAM", "", package = "VALERIE")
head(list.files(BAM))
#> [1] "SRR7103107_STAR_aligned_2ndPass_Aligned.sortedByCoord.out.bam"
#> [2] "SRR7103107_STAR_aligned_2ndPass_Aligned.sortedByCoord.out.bam.bai"
#> [3] "SRR7103235_STAR_aligned_2ndPass_Aligned.sortedByCoord.out.bam"
#> [4] "SRR7103235_STAR_aligned_2ndPass_Aligned.sortedByCoord.out.bam.bai"
#> [5] "SRR7103236_STAR_aligned_2ndPass_Aligned.sortedByCoord.out.bam"
#> [6] "SRR7103236_STAR_aligned_2ndPass_Aligned.sortedByCoord.out.bam.bai"
```

Running example data

The first step is to compute percent spliced in (PSI) values at each genomic coordinate spanning the alternative exons and flanking constitutive exons using **ComputePSI()** function. The object returned is a data frame of class *rehab*. Next, we plot the PSI values using **PlotPSI()** function: Splicing event type can be specified using *EventType* argument and the number of single cell groups can be specified using *Groups* argument whereas the location of output plots are specified in the *Plots* argument.

```r
# Compute PSI
PSI <- ComputePSI(SampleInfo = system.file("extdata/Sample_Info",
    "Sample_Info.txt", package = "VALERIE"), ExonInfo = system.file("extdata/Exon_Info",
    "Exon_Info.txt", package = "VALERIE"), BAM = system.file("extdata/BAM",
    "", package = "VALERIE"), MinCoverage = 10)

# Plot PSI (Output as shown in Figure 1)
PlotPSI(object = PSI, SampleInfo = system.file("extdata/Sample_Info",
    "Sample_Info.txt", package = "VALERIE"), ExonInfo = system.file("extdata/Exon_Info",
    "Exon_Info.txt", package = "VALERIE"), statistical.test = "wilcox",
    multiple.testing = "bonferroni", Plots = tempdir(), plot.width = 5,
    plot.height = 8, EventType = "SE", Groups = 2)
```

```r
# Check plot
output <- system.file("extdata/Plots", "1_SE_Plots_Mbp.pdf",
    package = "VALERIE")
knitr::include_graphics(output)
```

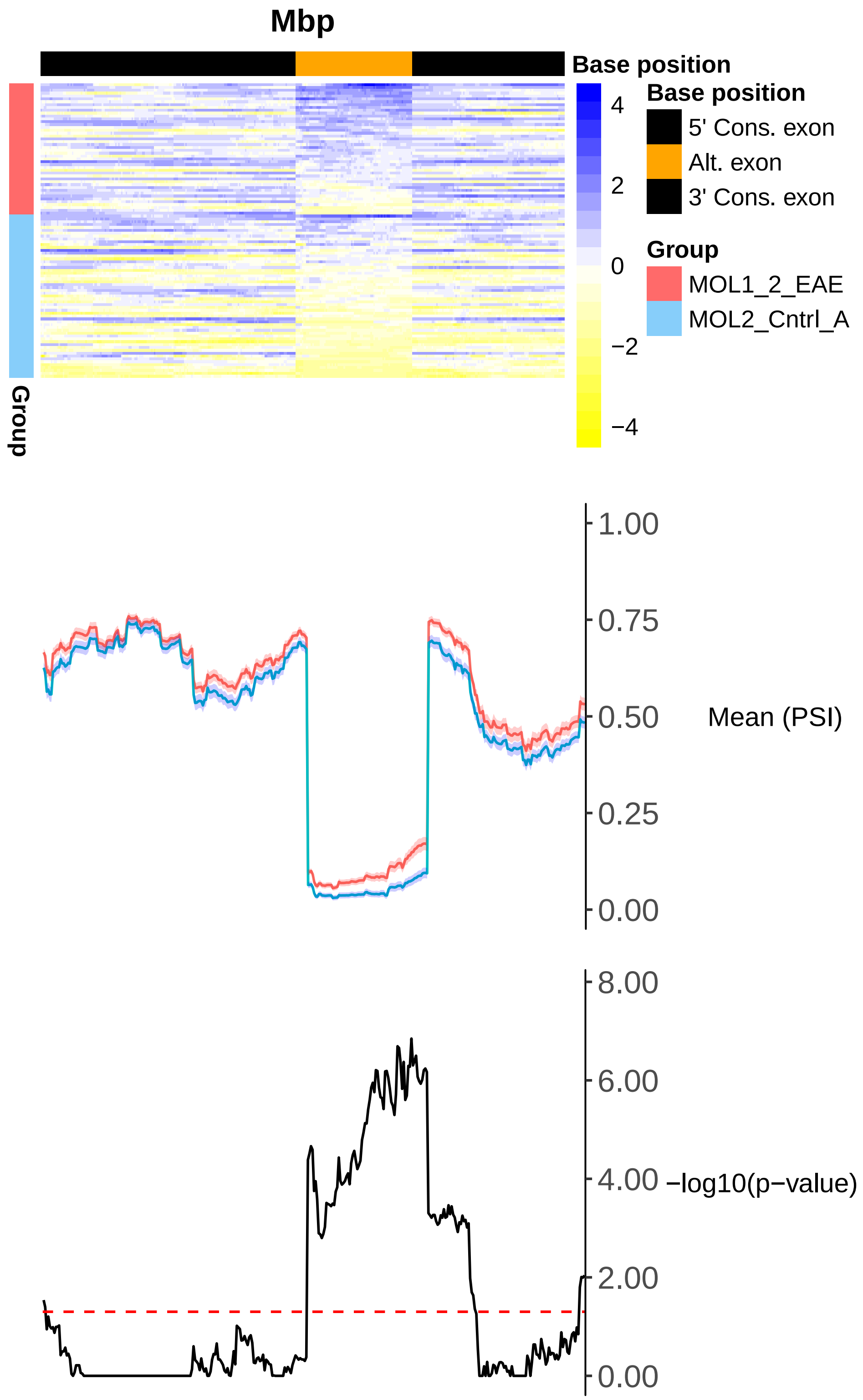

References

[1] Li, Z., et al., Pro-apoptotic effects of splice-switching oligonucleotides targeting Bcl-x pre-mRNA in human glioma cell lines. Oncol Rep, 2016. 35(2): p. 1013-9.

[2] Song, Y., et al., Single-Cell Alternative Splicing Analysis with Expedition Reveals Splicing Dynamics during Neuron Differentiation. Mol Cell, 2017. 67(1): p. 148-161 e5.
[3] Byrne, A., et al., Nanopore long-read RNAseq reveals widespread transcriptional variation among the surface receptors of individual B cells. Nat Commun, 2017. 8: p. 16027.
[4] Thorvaldsdottir, H., J.T. Robinson, and J.P. Mesirov, Integrative Genomics Viewer (IGV): high-performance genomics data visualization and exploration. Brief Bioinform, 2013. 14(2): p. 178-92.
[5] Falcao, A.M., et al., Disease-specific oligodendrocyte lineage cells arise in multiple sclerosis. Nat Med, 2018. 24(12): p. 1837-1844.
[6] Ramskold, D., et al., Full-length mRNA-Seq from single-cell levels of RNA and individual circulating tumor cells. Nat Biotechnol, 2012. 30(8): p. 777-82.
[7] Huang, Y. and G. Sanguinetti, BRIE: transcriptome-wide splicing quantification in single cells. Genome Biol, 2017. 18(1): p. 123.
[8] Katz, Y., et al., Analysis and design of RNA sequencing experiments for identifying isoform regulation. Nat Methods, 2010. 7(12): p. 1009-15.
