# Supplementary material for: VALERIE: Visual-based inspection of alternative splicing events at single-cell resolution
Source: PLoS Comput Biol. 2020 Sep 8;16(9):e1008195. doi: 10.1371/journal.pcbi.1008195 (PMC7500686; doi:10.1371/journal.pcbi.1008195)
Supplement: Attachment — Submitted filename: WenWX_VALERIE_PLoS Comput Biol_response_to_reviewers.docx [file pcbi.1008195.s007.docx]

**Detailed Responses to Reviewer’s Comments**

**Reviewer #1**

In this manuscript, Wen and colleagues describe a new tool for the visualization of alternative splicing events at single-cell resolution. They have named it VALERIE and they have made it available as an R package on CRAN. The authors have provided an example of the ability of VALERIE (compared to other platforms) to visualize an alternative splicing event at both single-cell and bulk levels using RNA sequencing data from the study by Song et al (Mol Cell 2017) on induced pluripotent stem cells, motor neuron cells and neural progenitor cells. Overall, VALERIE has several useful features that complement existing visualization platforms and is a valuable addition to the relatively small toolbox currently available for the visualization of alternative splicing events from RNA-seq data.

We thank the reviewer for these positive comments. We are glad that the reviewer appreciates the potential of VALERIE as a useful tool for the visualisation of alternative splicing events at single-cell resolution.

1. The authors have demonstrated the ability of VALERIE to visualize alternative splicing events and perform statistical tests using a single example of mutually exclusive exons (MXE) in the PKM gene in iPSCs and neuron cells. It would be interesting to see the visualization of at least another alternative splicing event (preferably a different type from MXE) either from the same study or from a different suitable study. Another example is shown in Figure S1, but this was specifically presented for data including very short reads.

We thank the reviewer for pointing this out. We demonstrated the application of VALERIE on additional alternative splicing events corresponding to exon-skipping events located on different locations at the 3’-end, 5’-end, and in the middle of the transcript. We incorporated these results in the revised manuscript found in Figure S5 and explained in the Result section from lines 178-189.

2. Line 82: The authors should specify which panel of Figure 1 refers to the image generated by VALERIE.

Figures 1C-E were generated by VALERIE. We clarified this in the text lines 136-138 and described in the Figures 1C-E legend. We did not specify Figures 1C-E at line 82 because we have yet to refer to Figure 1A or 1B. These have to be referenced in order.

3. In Figure 1 and Figure S1, it would be helpful to add a short text describing what the heatmap shows (i.e. PSI values) next to the colorbar.

The colour bar shows the scaled PSI values (z-scores) across rows of single cells. We described this in the figure legends for clarity instead of next to the colour bar to avoid overcrowding the plots.

4. In the Introduction, the syntax in line 10 should be corrected.

The syntax in line 10 was corrected.

**Reviewer #2**

The article by Wei Xiong Wen describes a novel tool for visualisation of alternative splicing events in single-cell RNA sequencing data. Alternative splicing is an important mechanism for the generation of proteomic diversity from the genome, and is critically overlooked in most single-cell RNA-sequencing studies. Tools that assist in the detection and visualisation of alternative splicing events from single-cell data are therefore, very welcome. The VALERIE R package enables visualisation of percent spliced in (PSI) in lieu of read coverage to enable visualisation of a number of single-cell samples in parallel, using BAM files as an input. The paper is concise, and the tool itself is useful – I have a number of relatively minor comments I would like to see addressed in a final publication.

We thank the reviewer for these positive comments and we would like to address the constructive comments in the following.

1. How can this tool be used in a discovery process? The authors show that it can potentially resolve alternative splicing events in individual genes, but it would be important that the authors demonstrate how the tool can be used in conjunction with others to identify alternative splicing events from a large dataset and rank them on the basis of supporting evidence. A graphical overview of the data processing would be helpful.

We thank the reviewer to bring out this important point. We provided the graphical overview to show how VALERIE can be involved in a discovery process in Figure S1B and described in the Design and Implementation section found in lines 88-91. A graphical overview of the data processing is also shown in Figure S1A.

VALERIE enables validation via visual inspection of alternative splicing events identified from the standard genome-wide analysis such as the differential analysis of distinct groups of single cells. Significant events that passed through the statistical cut-off criteria can be subjected to VALERIE to generate the graphical representation of multiple events simultaneously. Events from the visual inspection using VALERIE can be flagged in addition to, or as an alternative to, other approaches such as qPCR and smFISH. These candidate events can subsequently be prioritised for downstream functional studies.

2. It must be made clearer that this method appropriate primarily for plate-based methods such as Smart-seq2 which capture full-length sequence (although this is fragmented for sequencing) and not appropriate for e.g. 10x genomics libraries.

We noted this under the Availability and Future Directions section in lines 202-204.

3. Is there a hypothetical maximum number of cells the tool can cope with?

VALERIE reads in BAM files as the input, including the information of genomic coordinates corresponding to the splicing events.  VALERIE is fast and efficient in reading in BAM files. This is because we implemented the function that selectively reads regions corresponding to the input information of alternative splicing events. The limitation in terms of the maximum number of cells VALERIE can process is depended on the capacity of the processing computer. Therefore, there is no hypothetical maximum number of cells that VALERIE can process.

We evaluated the computation time needed to process single cells. To this end, we downloaded published data for the largest available single-cell alternative splicing analysis using RNA-seq data prepared with Smart-seq2 protocol. VALERIE required an average of 27.3s to process 2,000 single cells based on 10 repeated evaluations using the microbenchmark package. This evaluation was performed on an iMac with 3.5 GHz Quad-Core Intel Core i5 processer and 32 GB memory. We included this finding in the Results section of the revised manuscript found in lines 190-194.

4. Are there any mapping constraints for the method – i.e. are particular aligners more favourable than others. Is there any other pre-processing that might affect the outcome of the analysis?

VALERIE works directly with the BAM files. Therefore, it is conceivable that the most prominent factor which may affect the PSI profile is the mapping result. We used the STAR aligner for our analysis. HISAT2 is a recent mapping software with superior alignment speed compared to existing aligners. To evaluate the PSI profile generated by different aligners, we performed analysis on the same dataset using HISAT2. We observed differential PKM mutually exclusive exons 9 and 10 usage across induced pluripotent stem cells (iPSCs), motor neurons (MNs), and neural progenitor cells (NPCs) from BAM files generated from either HISAT2 or STAR.  We found a slightly higher level of mean PSI values and higher significant levels (lower p-values) from HISAT2 result compared to the STAR. This is may be in part attributed to the differences in sensitivity and specificity between the two aligners. We included this finding in the Results section lines 168-173 and Figure S3.

5. Similarly it would be essential to reflect on any coverage constraints for detection – e.g. how does the tool fare with genes at different expression levels (perhaps percentiles of overall expression levels) or indeed genes for which splicing events are located at the 3’, mid or 5’ of the transcript. I understand that some limitations here will be due to the biology/molecular biology preceding this analysis but it is important for potential users of the tool to understand potential limitations when thinking about this kind of analysis.

To address this question, we subsampled 50%, 25%, and 1% of the original read depth for each single cell to simulate different read depths and gene expression levels. As expected, we observed higher power to detect differential *PKM* mutually exclusive exons 9 and 10 usage at higher read depth as reflected by the clearer signal of mean PSI and significant level at genomic coordinates corresponding to the alternatively spliced exons. We included this finding in the Results section found in lines 175-178 and Figure S4.

We further selected three representative alternative splicing events from the same study to represent alternatively spliced exon located at the 3’-end, 5’-end, and in the middle of the transcript. These events were previously validated using qPCR. All alternative spliced exons showed differential usage across iPSCs, MNs, and NPCs by VALERIE. Notably, variable coverage was observed on the 3’-end and 5’end of constitutive exons which were the last and first exons of the transcripts, respectively. This observation was also reported in previous studies using long-read RNA-sequencing. VALERIE also revealed an alternative 3’ splice site (A3SS) on the 5’ constitutive exon (exon 5) based on changes in PSI values. We included this finding in the Results section found in lines 180-189 and Figure S5.

6. The main figure is quite basic and cluttered in appearance, panels C should be split into C, D and E and some additional explanation in the figure legend.

Panel C has been split into C, D, and E in the revised manuscript. Additional explanations were also included in the figure legend for clarity.

**Minor amendments**

Exon numbers were added to the main and supplemental figures for clarity.
